# Successful Endoscopic Ultrasound‐Guided Transgastric Drainage for Intra‐Abdominal Abscess Caused by Delayed Perforation After Gastric Endoscopic Mucosal Resection

**DOI:** 10.1002/deo2.70301

**Published:** 2026-02-21

**Authors:** Fumiaki Tanino, Akinori Shimizu, Taiki Nobuto, Yasuhiro Okuda, Yudai Takehara, Masaki Wakai, Tetsuro Hirano, Seiji Onogawa, Keiji Hanada, Shinji Tanaka

**Affiliations:** ^1^ Department of Gastroenterology Onomichi General Hospital Hiroshima Japan

**Keywords:** delayed perforation, endoscopic mucosal resection, endoscopic ultrasound, intra‐abdominal abscess, transgastric drainage

## Abstract

Delayed perforation after gastric endoscopic mucosal resection (EMR) is a rare but serious complication that can lead to intra‐abdominal abscess (IAA) formation. Efficacy of endoscopic ultrasound (EUS)‐guided drainage for IAA remains to be elucidated. A man in his 50s was admitted for EMR of hyperplastic polyps located in the gastric body. The procedure was performed uneventfully; however, on postoperative day (POD) 4, the patient developed a sudden fever accompanied by an elevation in inflammatory markers. Abdominal computed tomography (CT) revealed a localized area of increased fat attenuation, leading to a diagnosis of delayed perforation. The patient's condition temporarily improved with conservative therapy; however, fever and elevated inflammatory markers recurred on POD 17. CT demonstrated IAA requiring drainage. On POD 22, EUS‐guided transgastric drainage was performed using a nasobiliary drainage tube and double‐pigtail stent. After drainage, fever and inflammatory markers promptly alleviated; follow‐up CT demonstrated shrinkage of the abscess cavity. The clinical course was uneventful. EUS‐guided transgastric drainage could be a safe and effective therapeutic option for IAA secondary to delayed perforation after gastric EMR, particularly when conservative therapy fails and percutaneous drainage is not feasible.

## 1 Introduction

Endoscopic mucosal resection (EMR) and endoscopic submucosal dissection (ESD) are widely established as minimally invasive treatments for benign gastric lesions and early gastric cancer. However, complications such as bleeding and perforation can occur. Delayed perforation occurs in approximately 0.4% of cases and may follow a severe clinical course [1]. Although surgery and conservative management have been performed for delayed perforation after endoscopic resection (ER), effective drainage is required when conservative therapy fails. Percutaneous drainage is a standard treatment, but it may be difficult depending on anatomical limitations. Endoscopic ultrasound (EUS)‐guided transgastric drainage is a minimally invasive option for intra‐abdominal abscess (IAA); however, its role in IAA after ER remains unclear. We report effective use of EUS‐guided transgastric drainage for IAA following delayed perforation after EMR.

## 2 Case Report

**FIGURE 1:**
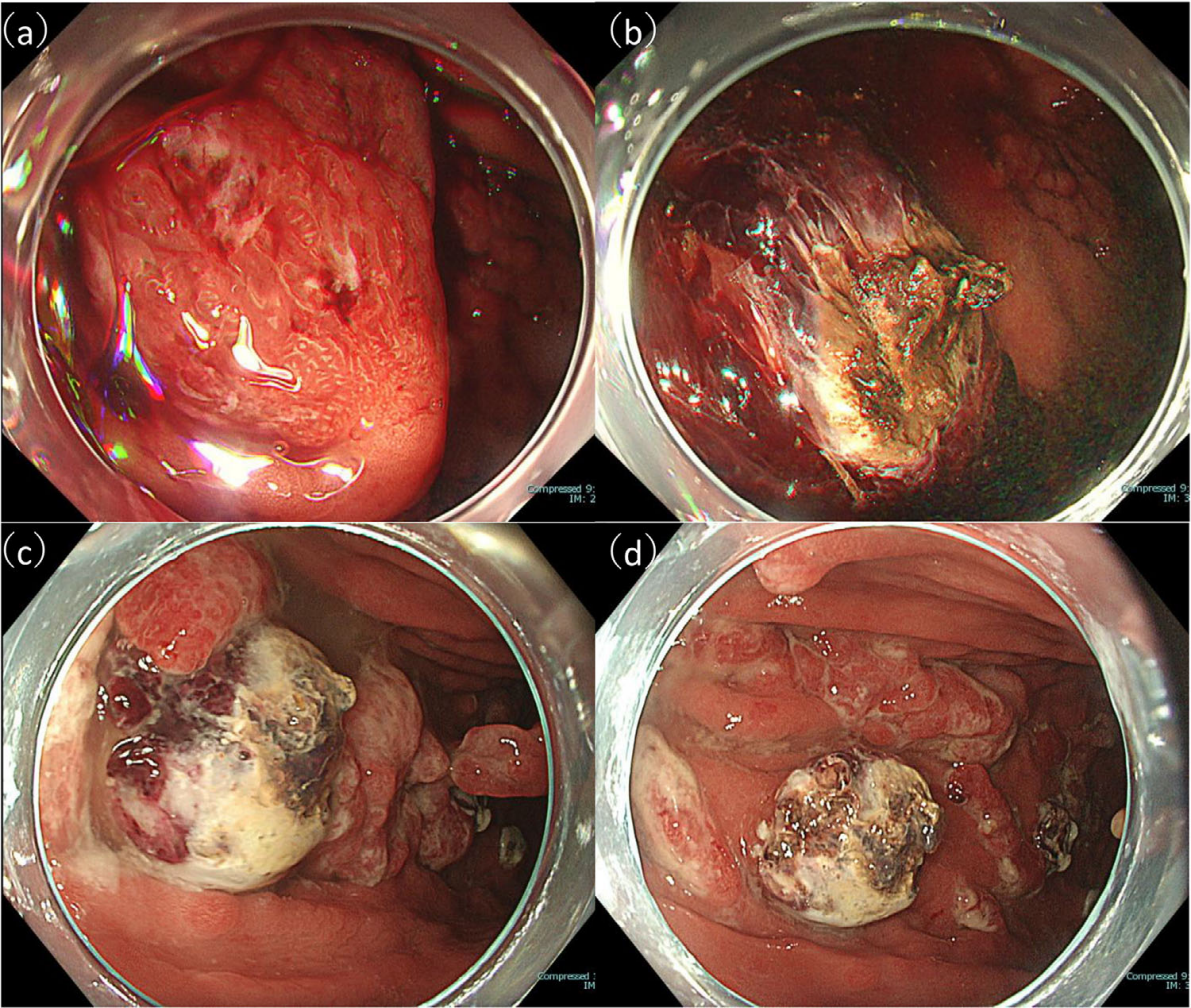
(a) Hyperplastic polyp located on the greater curvature of the upper gastric body. (b) Additional coagulation hemostasis was performed for bleeding at the EMR ulcer. (c): Follow‐up EGD on the following day showing a visible vessel at the ulcer base, requiring further coagulation hemostasis. (d): Endoscopic image of the ulcer base after additional coagulation hemostasis. No apparent perforation is seen. EGD, esophagogastroduodenoscopy; EMR, endoscopic mucosal resection.

**FIGURE 2:**
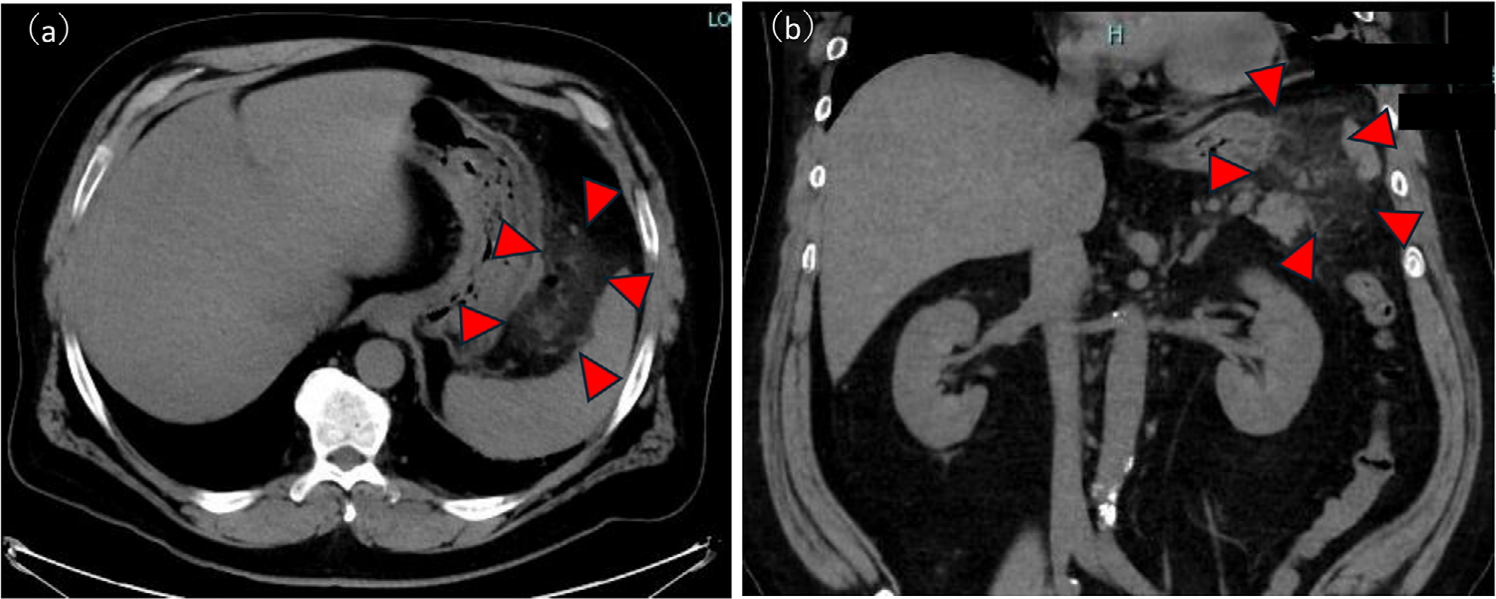
Computed tomography image obtained on postoperative day 4, showing free air and increased fat density around the stomach (red arrowhead), consistent with delayed perforation. (a) Axial view (b) Coronal view.

**FIGURE 3:**
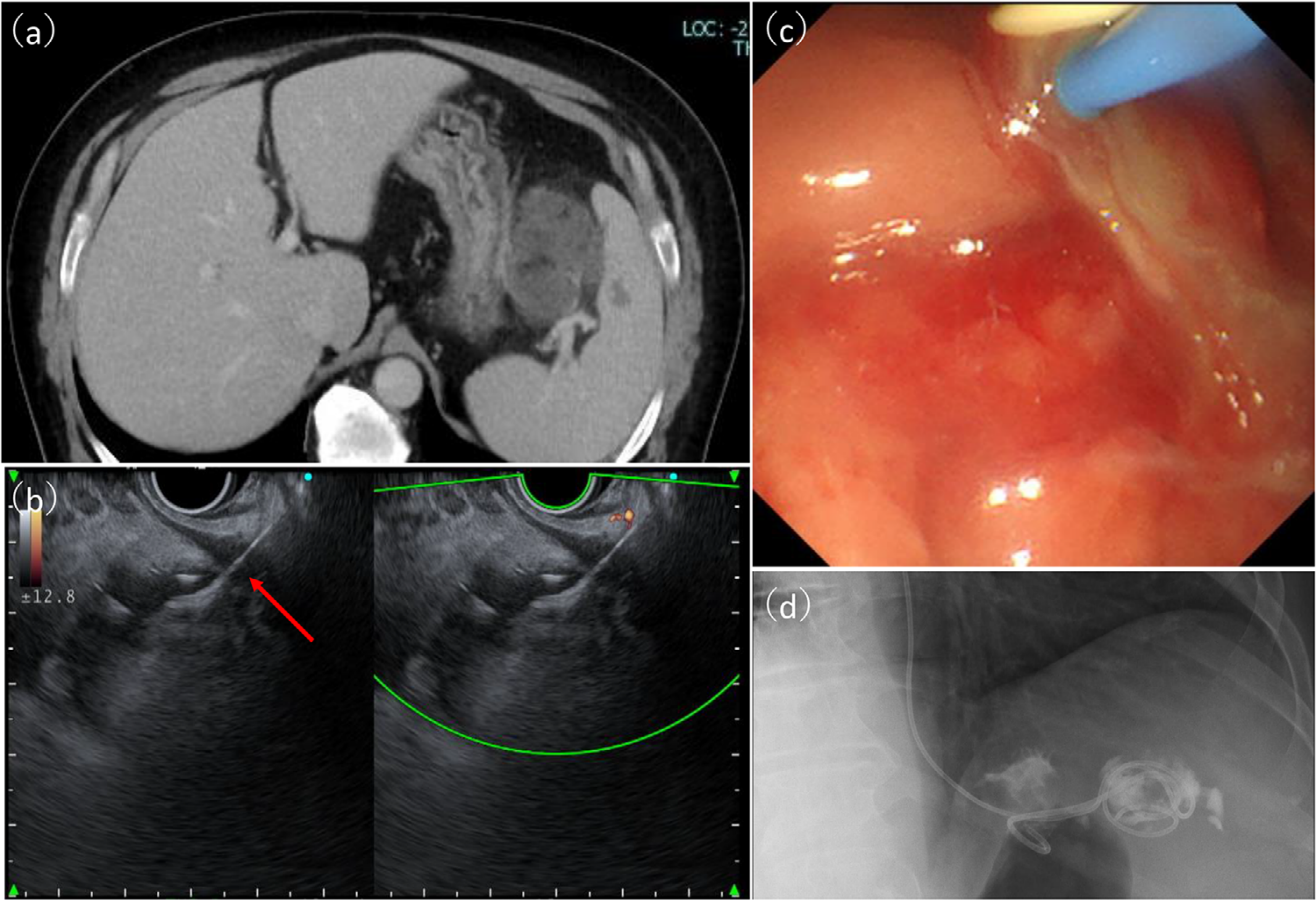
(a) Computed tomography image showed the increasing size of the abscess. (b) Endoscopic ultrasound image showing the puncture (red arrow) of the fluid collection using a 19‐G needle. (c) Drainage procedure showing the outflow of turbid, viscous, purulent material from the abscess cavity. (d) Placement of a 6Fr nasobiliary drainage tube and a 7Fr double‐pigtail stent in the abscess cavity.

**FIGURE 4:**
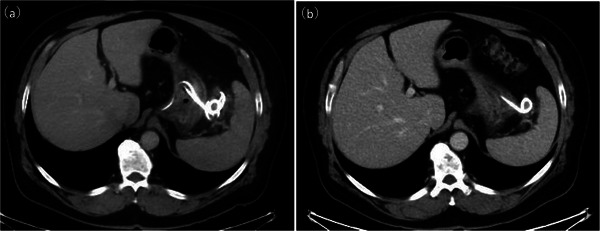
(a) CT image obtained 1 week after EUS‐guided drainage, showing a reduction in the size of the abscess cavity. (b) CT image obtained 1 month after EUS‐guided drainage, showing near‐complete resolution of the abscess cavity. CT, computed tomography; EUS, endoscopic ultrasound.

A man in his 50s with severe obesity (body mass index, 37.2) and a medical history of asthma, hyperuricemia, and diabetes mellitus presented to a local clinic with black stools and was diagnosed with iron‐deficiency anemia. He presented to our hospital for further evaluation. Esophagogastroduodenoscopy (EGD) was performed to investigate the cause of the anemia. EGD revealed multiple pedunculated gastric hyperplastic polyps with contact bleeding. After discussing treatment options with the patient, gastric EMR was performed using a GIF‐H290 endoscope (Olympus, Japan) for one pedunculated hyperplastic polyp (40 mm) on the greater curvature of the upper gastric body (Figure 1a) and two pedunculated hyperplastic polyps (both 30 mm) on the greater curvature of the middle gastric body. A submucosal injection of normal saline mixed with indigo carmine was administered to achieve adequate mucosal elevation. Mucosal resection was performed using a 13‐mm stiff stainless‐steel snare (Captivator; Boston Scientific, USA) with the electrosurgical generator (VIO300D; ERBE, Germany) set to Endocut Q mode (effect 3, cut duration 2, cut interval 3). Several exposed vessels were observed on the ulcer base. Hemostasis was achieved using a hemostatic forceps (Coagrasper; Olympus, Japan) in soft Coag mode (90 W, effect 5) (Figure 1b). On postoperative day (POD) 1, follow‐up EGD revealed visible vessels at the EMR ulcer base in the upper body lesion. Additional hemostasis was performed, and no perforations were evident (Figure 1c,d). Oral intake was resumed on POD 2. On POD 4, stable vital signs and absence of any abdominal tenderness, rebound tenderness, guarding, and peritoneal signs were observed; however, fever (>38°C) developed. Laboratory data revealed elevated inflammatory markers (white blood cells [WBC], 16700/µL; C‐reactive protein [CRP], 19.5 mg/dL). Abdominal computed tomography (CT) revealed free air and fat stranding adjacent to the upper gastric body. Therefore, delayed perforation after EMR was diagnosed (Figure 2). As no peritoneal signs were observed and considering the stable clinical course, conservative therapy with fasting and antibiotic therapy with meropenem 3 g per day intravenously was administered, after consulting with the surgical team. Although the patient's condition temporarily improved, fever and elevated inflammatory markers (WBC, 45,000 /µL; CRP, 12 mg/dL) were noted on POD17. No peritoneal signs were observed; however, CT revealed worsening fat stranding and IAA formation (Figure 3a). Blood cultures grew *Parvimonas micra*. As no further improvement was expected with conservative therapy, drainage for IAA was considered necessary. On POD 20, EGD confirmed the absence of a visible perforation at the EMR ulcer base, and EUS revealed heterogeneous fluid collection (diameter, >50 mm) without significant blood flow signals along the planned puncture route. On POD 22, EUS‐guided transgastric drainage was performed using a linear echoendoscope and Endoscopic Ultrasound Processor (GF‐UCT260/EU‐ME3; Olympus, Japan) to visualize the abscess cavity. After identifying a safe puncture route under EUS guidance, fine‐needle aspiration of the abscess was performed using a 19‐gauge needle (Expect 19; Boston Scientific, USA). A 0.035‐inch guidewire (Jagwire Plus, Boston Scientific, USA) was inserted through the needle. Subsequently, a 6‐Fr nasobiliary drainage tube (Flexima ENBD Catheter, Boston Scientific, Japan) and 7‐Fr double‐pigtail stent (AdvanixJ; Boston Scientific, Japan) were deployed (Figure 3a–d). White viscous fluid was drained from the abscess cavity, and abscess cultures also yielded *P. micra* (Figure 3c ). The fever and inflammatory markers rapidly improved following drainage. On　POD 29, laboratory data showed improved inflammatory markers (WBC, 9000 /µL; CRP, 0.8 mg/dL). Contrast‐enhanced CT performed 1 week later confirmed a reduced abscess cavity (Figure 4a). The nasobiliary drainage tube was removed on POD 30. Oral intake was resumed on POD 32. Intravenous antibiotics were switched to a 2‐week course of oral amoxicillin. The patient was discharged on POD 41. Follow‐up contrast‐enhanced CT performed 1 month later showed near‐complete resolution of the abscess cavity (Figure 4b).

## 3 Discussion

This case demonstrated the successful EUS‐guided transgastric drainage of IAA caused by delayed perforation after gastric EMR. The proposed mechanisms of perforation include ischemic necrosis of the gastric wall caused by repeated electrocautery or coagulation during submucosal dissection, and mucosal injury caused by retained bile reflux in the stomach [2]. Previous studies have shown that longer cautery duration is associated with perforation, with significantly longer cautery times in perforation sites than in non‐perforated areas [3]. In this case, the lesion was a pedunculated hyperplastic polyp, located on the greater curvature of the upper gastric body, which is prone to fluid pooling. Profuse bleeding was observed from the polyp, and fluid accumulation in the visual field was observed; therefore, identifying the bleeding point was difficult. Consequently, the prolonged hemostatic procedure time was considered the cause of delayed perforation. Surgery is commonly required for delayed perforation accompanied by peritonitis; however, conservative therapy may be considered when peritoneal signs are absent. As some cases do not respond adequately to conservative therapy, percutaneous drainage is another option for localized abscesses; nonetheless, anatomical limitations (such as obesity or interposed organs) can create difficulty securing a safe puncture route. Thus, EUS‐guided transgastric drainage is a desirable option when conservative therapy fails, and it has become increasingly popular for managing various fluid collections and postoperative abscesses [4]. Several reports have described its effectiveness for abscesses secondary to delayed perforation after gastric or colonic ESD [5, 6, 7, 8]. In this case, although initial conservative therapy resulted in temporary improvement, IAA enlarged, and anaerobic infection with *P. micra* progressed. Therefore, abscess drainage was necessary. Percutaneous drainage was assessed using abdominal ultrasonography; However, given the obesity, the skin and abscess cavity were separated by a considerable distance, which raised concerns about needle instability. Furthermore, the abscess cavity was surrounded by the stomach, intestinal tract, and spleen, rendering a percutaneous approach unsafe. In contrast, the EUS‐guided approach offered a safer and more feasible access route because of a shorter puncture distance and fewer interposed organs. Additionally, real‐time EUS imaging allowed confirmation of vascular structures and facilitated secure puncture route planning. After consultation with the surgical team, EUS‐guided drainage was selected. Both internal and external drainage were performed. Internal drainage with a double‐pigtail stent provides continuous abscess decompression; however, it may be insufficient in abscesses containing highly viscous pus or necrotic debris because of stent occlusion. Therefore, combined drainage with adjunctive external drainage using a nasobiliary drainage tube has advocated to enable irrigation, drainage control, and microbiological assessment and has been reported to be effective in various fluid collection and postoperative abscesses [9]. Identification of *P. micra* in both blood and abscess cultures suggested hematogenous spread from the localized abscess and emphasized the importance of early and effective drainage. Although EUS‐guided drainage for IAA is minimally invasive, safe, and effective, it is associated with risks such as perforation, bleeding, and stent migration [10]. To our knowledge, this is among the few reported cases of successful EUS‐guided drainage of IAA after gastric EMR, thus expanding its indications beyond perforation after ER. This case highlights the importance of timely recognition of delayed perforation and the potential of EUS‐guided drainage as an alternative treatment strategy. EUS‐guided transgastric drainage can be applied as a safe therapeutic option for IAAs without peritoneal signs after ER, particularly when cases are unresponsive to conservative therapy. Further accumulation of similar cases is warranted to establish standardized indications and procedural safety.

## Author Contributions

**Fumiaki Tanino**: conceptualization, methodology, data curation, visualization, investigation, and writing. **Akinori Shimizu**: conceptualization, methodology, supervision, review, and editing. **Taiki Nobuto**: review and editing. **Yasuhiro Okuda**: review and editing. **Yudai Takehara**: review and editing. **Masaki Wakai**: review and editing. **Tetsuro Hirano**: review and editing. **Seiji Onogawa**: supervision, review, and editing. **Keiji Hanada**: conceptualization, methodology, supervision, review, and editing. **Shinji Tanaka**: review and editing.

## Funding

This research received no specific grant from any funding agency in the public, commercial, or not‐for‐profit sectors.

## Conflicts of Interest

The authors declare no conflicts of interest.
